# Relationships Among Maternal Epstein–Barr Virus Antibodies, COVID‐19, and Stress in Mothers up to 1‐Year Postpartum

**DOI:** 10.1002/ajhb.70289

**Published:** 2026-06-12

**Authors:** Emma Anastasi, Delaney J. Glass, Tiffany D. Pan, Eleanor Brindle, Beatrice Caffe, Caroline B. Smith, Amanda E. Kunkle, Maia A. Kent, Alexandra D. Navarrete, Christina D. W. Pace, Dan T. A. Eisenberg, Ryan M. Pace, Janet E. Williams, Sylvia H. Ley, Celestina Barbosa‐Leiker, Mark A. McGuire, Michelle K. McGuire, Courtney L. Meehan, Melanie A. Martin

**Affiliations:** ^1^ Department of Anthropology University of Washington Seattle Washington USA; ^2^ Center for Studies in Demography and Ecology University of Washington Seattle Washington USA; ^3^ Department of Anthropology University of Toronto‐St. George Toronto Ontario Canada; ^4^ Integrated Maternal and Child Health and Development, PATH Seattle Washington USA; ^5^ Department of Anthropology Washington State University Pullman Washington USA; ^6^ Elson S. Floyd College of Medicine Washington State University Spokane Washington USA; ^7^ Department of Medicine Oregon Health & Sciences University Portland Oregon USA; ^8^ Margaret Ritchie School of Family and Consumer Sciences University of Idaho Moscow Idaho USA; ^9^ College of Nursing University of South Florida Tampa Florida USA; ^10^ Microbiomes Institute, University of South Florida Tampa Florida USA; ^11^ Department of Animal Veterinary and Food Sciences, University of Idaho Moscow Moscow Idaho USA; ^12^ Department of Epidemiology Tulane University Celia Scott Weatherhead School of Public Health New Orleans Louisiana USA; ^13^ College of Nursing Washington State University Health Sciences Spokane Spokane Washington USA

**Keywords:** COVID‐19, Epstein–Barr virus, immunomodulation, infant care, maternal infection, PSS‐10, stress

## Abstract

**Objectives:**

Both psychosocial stress and current infection can increase the likelihood of reactivation of latent Epstein–Barr virus (EBV). Pregnancy and infection with SARS‐CoV‐2 can be stressful and modulate immune function. However, it is unknown how SARS‐CoV‐2 infection and stress further interact among postpartum women to affect EBV antigen and antibody expression.

**Methods:**

We examined associations among COVID‐19 diagnosis, self‐reported stress, and EBV immunoglobulin G (IgG) in previously collected data from 45 breastfeeding US women (1–18 months postpartum) during the height of the COVID‐19 pandemic. Between June 2020 and March 2021, stress survey data and capillary dried blood HemaSpot samples were collected from 25 SARS‐CoV‐2 infected and 20 non‐infected participants at multiple time points over the course of 60 days. Samples (*N* = 104) were later assayed for EBV IgG. Statistical methods were pre‐registered, and relationships among SARS‐CoV‐2 infection, stress, and EBV IgG were analyzed via mixed‐effects regression models, adjusting for time postpartum and time elapsed since enrollment.

**Results:**

There was a statistically significant association between self‐reported stress and EBV IgG levels, with higher average stress associated with higher average EBV IgG. EBV IgG relative concentration did not vary independently with COVID‐19 positivity status or the number of COVID‐19 symptoms.

**Conclusions:**

Results suggest that EBV IgG relative concentrations were elevated across sample participants by stress, but not by the relatively mild cases of COVID‐19 the mothers experienced.

## 1 Introduction

Epstein–Barr virus (EBV) is a pervasive pathogen that can cause acute illness and persist in a latent state after initial exposure. EBV is transmitted through direct contact with infected saliva or contact with contaminated objects. About 90% of people are exposed to EBV by the time they reach adulthood (Haeri et al. 2010). Most people are infected in childhood and do not experience symptoms, but exposures during young adulthood are more likely to result in infectious mononucleosis, with symptoms including fever, sore throat, swollen lymph nodes, and fatigue (CDC 2022). After initial exposure, the viral genome generally remains latent in the body, with only a few genes being expressed. Viral reactivation can occur after latency, in which the full viral genome is expressed, and this can be triggered by many immunomodulating factors, including pregnancy, concurrent infections, and stress (Glaser et al. 2005; Haeri et al. 2010). The association between stress and EBV viral load has been observed and experimentally demonstrated for acute stressors (e.g., exam conditions and instances of racial discrimination) and proxy stress measures such as counts of depressive symptoms, perceived stress scores, and salivary cortisol levels (Brook et al. 2017; Christian et al. 2012; Sausen et al. 2021; Zhu et al. 2013).

Much of the research supporting an association between EBV antibody elevation and stress focuses on stress as a trigger of EBV reactivation, as opposed to illness after initial exposure. Reactivations are most often identified when patients experience symptoms such as fatigue, fever, and inflammation of the throat or lymph nodes, and an EBV reactivation is subsequently confirmed through an antibody blood test (CDC 2022). The presence or absence of antibodies against various EBV‐specific antigens can be used to distinguish reactivation from initial acute infection or simply a history of contact with the virus (Haeri et al. 2010). One of the antibodies most often used as an indicator of past EBV infection, and sometimes as a biomarker for immune modulation, is anti‐EBV viral capsid antigen immunoglobulin G (VCA IgG). VCA IgG antibodies are expressed after initial infections, symptomatic or not, and are also expressed during a reactivation (Haeri et al. 2010). Anti‐VCA immunoglobulin M (VCA IgM), anti‐early antigen IgG (EA IgG), and anti‐nuclear antigen immunoglobulin G (NA IgG) are variously present during acute initial infections, reactivations, or when the virus is latent, and so can be used to distinguish between these viral states (Haeri et al. 2010). Even though anti‐EBV VCA IgG titer does not individually distinguish between reactivation and presence of latent EBV, fluctuations in EBV VCA IgG can indicate immunomodulation. Relatively higher anti‐EBV VCA IgG levels have been interpreted as an indirect sign of suppressed or dysregulated cell‐mediated immunity, with a permitted increase in EBV viral load leading to a subsequent increase in anti‐EBV IgG production, while lower anti‐EBV IgG titers are interpreted as a sign of improved immunity (Christian et al. 2012).

The COVID‐19 pandemic provides a particularly interesting context in which to study EBV expression and reactivation. Patients with concomitant EBV reactivation and COVID‐19 are more likely to experience severe COVID‐19 signs and symptoms, especially high fevers (Chen et al. 2021). Other research has suggested that EBV reactivation may contribute to “long COVID” symptoms (Gold et al. 2021). In addition, while any infection or illness may be emotionally stressful or have immunomodulatory effects, people infected with SARS‐CoV‐2 in the first year of the pandemic also contended with the stress of a novel disease and social upheaval. Thus, SARS‐CoV‐2 infection during the height of the pandemic could have influenced immune suppression of EBV, antigen expression, and the risk of reactivation through multiple pathways.

New mothers with COVID‐19 during this time may have also experienced additional risks due to immunomodulating effects of postpartum recovery, lactation, and new maternal stressors. Even though the immune function of these mothers could have been influenced by coinfection with SARS‐CoV‐2, stress, and pregnancy‐related factors, we do not yet know which of these factors may have the largest influence on immune system function or the risk of EBV reactivation. Only a few studies have focused on immune function and EBV infection during pregnancy, and fewer still have focused on the postpartum period. One previous study found that EBV VCA IgG expression was highest in the first and second trimesters of pregnancy, lowest in the third trimester, then nearly as high during postpartum recovery as during the first trimester (Christian et al. 2012).

This exploratory study investigated associations among COVID‐19, stress, and EBV VCA IgG (hereafter EBV IgG) concentration using a subset of survey data and capillary dried blood samples previously collected from SARS‐CoV‐2 infected and non‐infected mothers (Martin et al. 2022). Nearly all mothers recruited into the original study were breastfeeding at the time of sample and data collection, which occurred early in the pandemic (June 2020–March 2021) when many social restrictions were still in place, and many uncertainties related to COVID‐19 remained. While SARS‐CoV‐2 infected mothers may have had higher risk of EBV reactivation, all participants faced variable stressors from social isolation, childcare challenges, and health anxiety, which may have further influenced overall immune function, EBV IgG expression, and the likelihood of potential EBV reactivation. Previous studies have consistently found that self‐isolation during the pandemic was especially challenging for families with young children who struggled to balance work, childcare, health and safety precautions, and finances (Fong and Iarocci 2020; Spinelli et al. 2020). Without access to support from other adults, parents experienced high stress which affected their mental health and ability to support their children (Fong and Iarocci 2020; Spinelli et al. 2020).

Preliminary analyses, hypotheses, and candidate models for this exploratory analysis were pre‐registered at Open Science Framework (Anastasi and Martin 2024). We hypothesized that EBV IgG would be higher in association with COVID‐19 infection and stress in independent models. We proposed to test for interactive effects between SARS‐CoV‐2 infection and stress and a moderating effect of symptoms in two other candidate models. In this study, we further describe possible environmental stressors and participant stress using survey questions related to infant feeding, sleep, activities within and outside the home, and a modified version of the 10‐question Perceived Stress Scale (PSS‐10) (Cohen et al. 1983).

## 2 Materials and Methods

### 2.1 Data Collection

Data for this study were collected between June 2020 and March 2021 as part of a multi‐site, collaborative study aimed at examining maternal and infant health outcomes and changes to milk composition following maternal COVID‐19 (Martin et al. 2022; Pace et al. 2021). Participants were recruited locally at participating institutions (Tulane University; University of Idaho; University of Washington; Washington State University) and nationally through social media advertising. The study recruited a total of 37 breastfeeding and non‐breastfeeding SARS‐CoV‐2‐infected mothers (COVID+), enrolled within 7 days of a self‐reported positive PCR test, and a comparative group of 27 mothers with no known SARS‐CoV‐2 infection or exposures (COVID−). COVID+ mothers were interviewed by phone at enrollment and Days 2–6, 7, 14, 21, 30, and 60 after enrollment; and COVID− mothers at enrollment and Days 1, 7, 21, and 60 after enrollment. Interviews included a one‐time household and demographic survey, and repeated surveys on COVID‐19 symptoms, household activities, infant care and feeding, and completion of the PSS‐10 (Cohen et al. 1983). PSS‐10 questions were rephrased to ask about feelings over the last week instead of over the last month (see Supporting Information Section 2).

Participants self‐collected maternal and infant capillary dried blood samples using HemaSpot (HS) collection devices (Spot On Sciences, San Francisco). HS absorbent paper blades are enclosed in a plastic case with a built‐in desiccant, enabling easy and safe self‐collection and storage. Samples were collected on Days 7, 14, 21, and 60 from the COVID+ group, and Days 7, 21, and 60 from the COVID− group. At the start of the study, mothers were between 30 days and 1.5 years postpartum.

HS samples collected from mother‐infant dyads were previously assayed for anti‐SARS‐CoV‐2 IgG and IgA antibodies at the University of Washington Center for Studies in Demography and Ecology Biodemography Lab (Martin et al. 2022). Here, we analyzed results of EBV IgG assays conducted on a subset of maternal samples with sufficient remaining blood volume and relatively equal representation across COVID‐19 status groups: 25 COVID+ participants (61 samples) and 20 COVID− participants (42 samples). All of these 45 participants were breastfeeding during data collection. Infant HS samples were not assayed for EBV IgG because initial EBV exposure in the United States usually occurs around 5 to 10 years old (CDC 2022).

### 2.2 EBV IgG Assay Methods

EBV IgG concentration was analyzed using a qualitative commercial enzyme immunoassay kit (Diamedix Immunosimplicity EBV‐VCA IgG kit #720‐600; Diamedix, Miami Lakes, FL), previously validated for quantitative EBV IgG measurement from protein card DBS samples (Eick et al. 2016) and further adapted for use with the HS devices (see Section 1.1 of Supporting Information). Our adapted protocol estimates resulting quantitative EBV concentrations in arbitrary units (AU) because our standard curve was created from a low positive control of arbitrary concentration. All samples, standards, and controls were tested in duplicate. The inter‐assay CVs based on the kit‐supplied low positive control and cutoff calibrator were 9.14 and 17.68, respectively. The mean intra‐assay CVs for three plates were 7.57%. All samples were within the limits of quantification (see Section 1.1 of Supporting Information). All plates passed quality control metrics from the published assay protocol (Eick et al. 2016; Supporting Information Section 1). Due to sample and resource limitations, it was not possible to rerun samples with inter‐duplicate CVs > 15% (*n* = 15) per standard lab protocols.

### 2.3 Analytical Methods

As described in the preregistration, candidate models use EBV IgG relative concentrations (AU) as the main outcome variable. While these concentration values are measured in AU, they are internally consistent and vary continuously on a linear scale. We used all results from all samples in final analyses after conducting a sensitivity analysis that demonstrated no substantial differences in results when excluding sample results with CVs > 15% (see Table S5). The models use enrollment group (COVID+/−) as a proxy of COVID‐19 infectious status, in part because many participants were vaccinated against COVID‐19 during the course of follow‐up, which likely influenced the outcomes of subsequent anti‐SARS‐CoV‐2 antibody blood tests. Participants were assigned to COVID+ at enrollment if they reported receiving a positive PCR test. After enrollment, 89% of COVID+ participants tested positive by antibody blood testing. While the preregistered analysis plan proposed use of PSS scores at each interview, due to differences in survey collection between the infected and non‐infected groups, our final models used participant mean PSS scores as an independent predictor of overall stress during the study.

All analyses were performed in R v4.4.1 (R Core Team 2023), using the *lme4* package (Bates et al. 2015) for mixed effect modeling and *ggplot2* (Wickham 2016) and *visreg* (Breheny and Burchett 2017) packages for data visualization. We evaluated four mixed‐effects linear regression models to explore the potential effects of COVID‐19 status and stress on average EBV IgG levels (see Table 2 for all variables included in models). While EBV IgG would be expected to acutely rise and then fall with acute infection in COVID+ participants, we chose linear mixed effects modeling because models included both COVID+ and COVID− participants, and the association between stress and EBV IgG was expected to be approximately linear.

All models included participant as a random intercept to account for multiple observations per participant, and survey day (as a measure of time since enrollment) and number of days postpartum as covariates, since it is established that EBV IgG levels fluctuate during pregnancy and take some time during postpartum recovery to return to baseline (Christian et al. 2012; Haeri et al. 2010). Our baseline model (Model 1) included COVID‐19 status as the main predictor variable. Model 2 added PSS scores, and Model 3 also included an interaction term between COVID‐19 status and PSS scores. Finally, Model 4 included the number of COVID‐related symptoms reported at sampling, as a proxy of infection severity. We use 95% confidence intervals to reflect uncertainty in variable coefficients, additionally noting *p*‐values below 0.05, and we use AIC values to compare model fits.

## 3 Results

### 3.1 Participant and Household Characteristics

Participants represent a white, middle class, highly educated population (Table 1). Participants reported household income brackets with breaks at $20 000, $35 000, $50 000, $75 000, and $100 000. Approximately 58% of participants reported a household income of $75 000 or higher, with the median household income in the United States for 2021 reported as $70 784 (Table 1) (Semega and Kollar 2022). Most participants self‐identified as White, Non‐Hispanic (84%), with the rest identifying as White, Hispanic (9%), Black/African American (1%) or Other (6%).

**TABLE 1:** Participant characteristics for COVID+ and COVID− groups from survey responses.

	Overall (*n* = 64)		COVID+ (*n* = 37)		COVID‐ (*n* = 27)	
	Mean ± SD Or proportion	Range	Mean ± SD Or proportion	Range	Mean ± SD Or proportion	Range
Maternal age (years)	32 ± 4	24–40	32 ± 4	24–40	32 ± 4	26–38
Maternal height (in.)	65 ± 3	60–73	66 ± 3	62–73	65 ± 3	60–72
Maternal weight (pounds)	172 ± 46	112–350	171 ± 41	112–260	173 ± 54	119–350
Maternal parity^a^	2 ± 1	0–5	2 ± 1	0–4	2 ± 1	1–5
Time postpartum at enrollment (days)	210 ± 148	17–630	214 ± 152	17–630	209 ± 145	24–546
Maternal ethnicity—White, non‐Hispanic	84%	—	83%	—	85%	—
Maternal education—College degree	69%	—	61%	—	78%	—
Household income > $75 K	59%	—	53%	—	66%	—

*Note:* Proportions given for most common category among categorical variables.

^a^ One participant included in this descriptive analysis breastfed an enrolled infant, but did not gestate the infant. This participant's parity is zero. This participant along with 21 other participants did not have sufficient sample remaining for EBV assay and their data are not included in EBV regression models.

In initial enrollment interviews nearly all (97%) of COVID+ mothers reported taking measures to protect their infants from infection during feedings (nursing or bottle feeding). This included washing their hands (30%) or breasts (0.5%) before feeding, wearing a mask while feeding (27%), or exclusively having other household members feed their infants pumped milk (9%). At 3 weeks postenrollment, however, only 0% and 3% of mothers continued to mask or wash hands/breasts for feedings, respectively, and none were having their infants fed by others exclusively. These percentages remained the same 2 months after enrollment. In contrast, only 11% of COVID− mothers reported any of these protective behaviors at enrollment, and by 3 weeks, no COVID− mothers reported these behaviors. Across groups, reported breastfeeding frequency ranged from 0 to 18 times per day within a 24‐h period (mean = 6 ± 4 SD times).

Only 7% of COVID+ mothers reported isolating themselves from their infants and other household members for several days after their initial positive COVID‐19 test. By the 2nd week of follow‐up, no mothers were isolating themselves from their infants. Across both groups, most infants (76%) either slept in a crib in the parent's room (36%), in their own room (37%), or both (3%). Twenty‐four percent of mothers reported that their infants slept in their bed, with 29% of COVID− mothers bed sharing, as compared to 20% among the COVID+ mothers. These proportions did not change substantially over time.

At enrollment, 33% of all participants were leaving home regularly for work and this increased to 48% at the two‐month follow‐up. These percentages differed in the expected direction between COVID+/− mothers (35% and 56% for COVID+ at enrollment and two‐month follow‐up, respectively; 37% and 44% for COVID‐). Similarly, at enrollment, 31% of mothers were leaving home for community activities—including family gatherings, church, social gatherings, and visiting relatives—which increased slightly to 38% after 2 months. These percentages also differed between groups (5% and 36% for COVID+ within 7 days of enrollment and two‐month follow‐up, respectively; 18% and 41% for COVID‐ mothers).

### 3.2 PSS Responses

**FIGURE 1:**
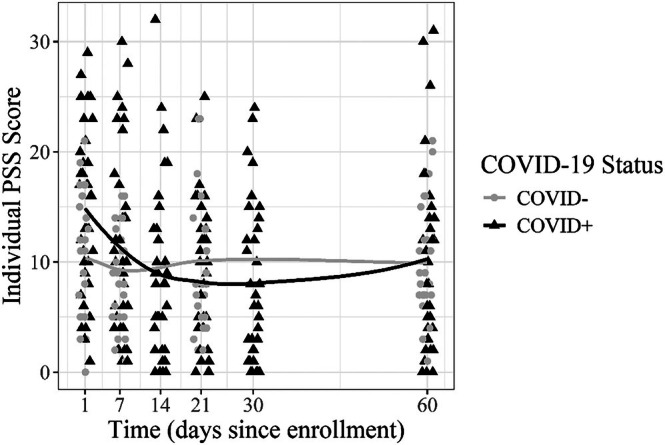
Perceived stress screening (PSS‐10) scores over time for COVID+ and COVID− mothers PSS‐10 scores per participant per survey day (1, 7, 14, 21, 30, and 60 says after study enrollment). Each survey score equals the sum of answers to the 10 screening questions, scored from 0 (“*never*”) to 4 (“*very often*”). COVID‐ mothers represented by gray circles; COVID+ mothers represented by black triangles (*N* participants = 64; *n* surveys = 315).

The PSS‐10 screening asked participants about thoughts and feelings related to stress and their ability to deal with it (see Tables S1–S3). The questionnaire includes 10 questions, and participants answer between 0 and 4 for each, with 4 representing the highest frequency of experiencing a given feeling. Scores are then summed (as described in Section 2 of Supporting Information). In the original PSS scale (which references stress perception over the last month), scores from 0 to 13 are categorized as “low” stress, scores from 14 to 26 are “moderate” and from 27 to 40 are “high.” Applying the same interpretation to our adapted scale (though thresholds have not been validated for perceptions over the last week), most participants scored “low” or “moderate” on the PSS‐10 across follow‐up periods, with only 3.6% of surveys scored as “high.” Given the limited variation in high stress scores as interpreted from the original scale, we modeled PSS‐10 scores continuously rather than categorically, which is also the recommended approach. The most frequent responses to individual PSS‐10 questions across participant follow‐up were “Never,” “Almost Never,” and “Sometimes.” The highest average responses were reported for the following questions: “In the last week, how often have you been upset because of something that happened unexpectedly?”; “In the last week, how often have you felt nervous and “stressed”?”; “In the last week, how often have you been angered because of things that were outside of your control?” Figure 1 displays PSS‐10 scores from all participants across all time points, with higher average scores among the COVID+ group at enrollment, decreasing to be more similar to the COVID‐ group within 2 weeks.

### 3.3 COVID‐19, EBV, and Stress (Preregistered Analysis)

**FIGURE 2:**
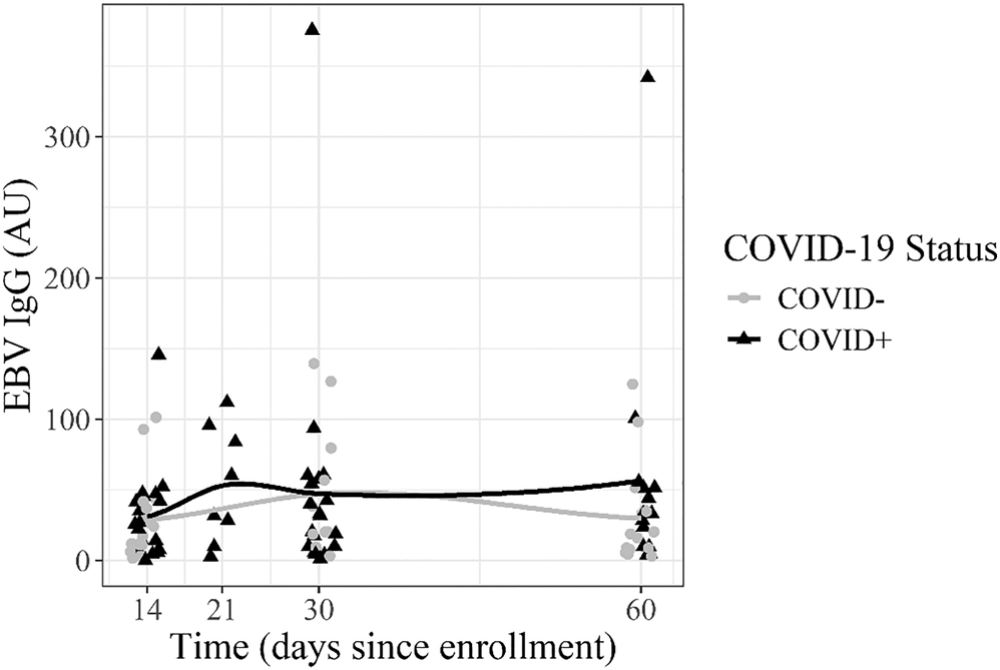
EBV IgG relative concentration (AU) over time for COVID+ and COVID− mothers scatterplot of Epstein–Barr virus immunoglobulin G (EBV IgG) relative concentration (in arbitrary units) across blood sample collection days (14, 21, 30, and 60 days after enrollment). COVID‐ mothers represented by gray circles; COVID+ mothers represented by black triangles. *N* participants = 45; *n* samples = 104.

EBV IgG relative concentrations (AU) were right skewed with a few high outliers (mean 40.58 ± 55.71 AU), with higher mean concentrations observed among COVID+ versus COVID‐ mothers (see Supporting Information Section 3 and Table S4). While mean EBV IgG was nominally higher among COVID+ versus COVID− mothers in the first month of follow‐up (Figure 2), patterns were similar across both groups. While the mean number of COVID‐19 symptoms reported was higher among COVID+ versus COVID− mothers, asymptomatic COVID+ mothers had higher mean EBV IgG concentrations (83.55 ± 115.17 AU) as compared to those reporting symptoms (e.g., 20.66 ± 2.51 AU for those reporting the maximum of seven symptoms) (Table S4).

Across follow‐up, one participant exhibited higher EBV concentrations and PSS‐10 scores than all other participants, sufficiently high to be considered an outlier (visible in Figure 2). Excluding this participant from analyses changed model coefficients and confidence intervals but not overall conclusions, so this participant was ultimately included in analyses (see Table S7).

**FIGURE 3:**
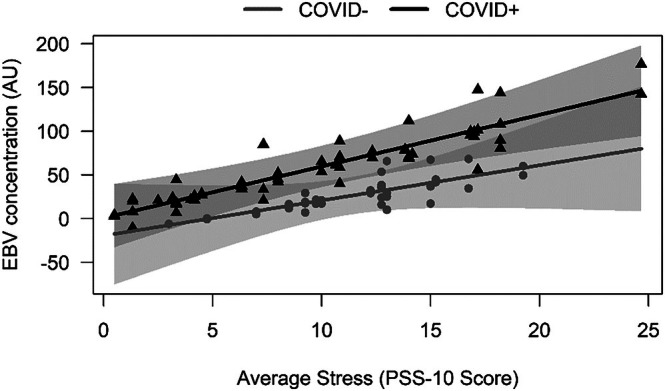
Predictive effect plot of the interaction of COVID‐19 and stress on EBV scatterplot of the association of stress (PSS score) with EBV IgG relative concentration in arbitrary units (AU), separated by COVID‐19 status, with COVID+ data shown in black triangles and COVID− shown in gray circles. Shaded regions represent 95% confidence intervals.

Few group differences in EBV concentrations persisted once adjusting for other covariates in linear mixed‐effects models (Anastasi and Martin 2024). In our baseline model (Table 2, Model 1), EBV IgG relative concentrations (AU) did not vary consistently in relation to COVID‐19 status. In Model 2, PSS score was independently associated with EBV IgG relative concentration (AU), and its inclusion in the model improved model fit as assessed by AIC. In Model 3, the interaction of stress (PSS‐10) and COVID‐19 status was not significantly associated with EBV IgG relative concentration, with wide overlapping confidence intervals, though model fit improved (Table 2, Model 3). The coefficient for COVID‐19 status indicated higher EBV on average for infected mothers, but confidence intervals included zero in all four models. Associations with time postpartum and time elapsed since enrollment were negligible and uncertain in all models. Per our preregistered analysis plan, we ran an additional model including COVID‐19 symptoms based on the best‐fitting prior alternate model (Table 1, Model 4). This resulted in the strongest model and suggested an overall trend contradicting our expectations, in which higher COVID‐19 symptoms were associated with lower EBV IgG relative concentrations (AU), on average. Figure 3 highlights the predictive effect of stress on EBV concentration within the COVID‐19 status groups based on model 4. Neither the direction nor significance of estimates changed substantially across models when EBV IgG was log‐transformed (Table S6).

**TABLE 2:** Mixed effects linear model fitted coefficients with EBV IgG as the outcome variable.

	Model 1	Model 2	Model 3	Model 4
COVID‐19 (+/−)	17.72 (−14.69, 50.15)	24.24 (−3.68, 52.16)	0.14 (−68.05, 68.33)	20.17 (−48.99, 89.33)
Days postpartum	0.11 (−0.01, 0.23)	0.10 (−0.01, 0.20)	0.09 (−0.01, 0.20)	0.06 (−0.04, 0.17)
Days since enrollment	0.03 (−0.18, 0.24)	0.02 (−0.18, 0.23)	0.02 (−0.18, 0.23)	0.02 (−0.18, 0.23)
Stress (avg. PSS score)	—	**5.22 (2.64, 7.80)** ***p* = 0.0004**	3.58 (−1.37, 8.53)	4.03 (−0.76, 8.83)
Stress × Covid‐19	—	—	2.25 (−3.57, 8.07)	1.88 (−3.75, 7.50)
Number of symptoms	—	—	—	−6.97 (−14.42, 0.49)
AIC	1030.33	1016.88	1014.26	1008.66

*Note:* 95% confidence intervals included in parentheses. All results are non‐significant unless *p‐*value indicated in bold (*n* participants = 45; *n* observations = 104). Coefficients and confidence intervals are rounded to two decimal places to make it clear when confidence intervals for small coefficients cross zero. Rounding does not necessarily indicate the extent of precision.

**FIGURE 4:**
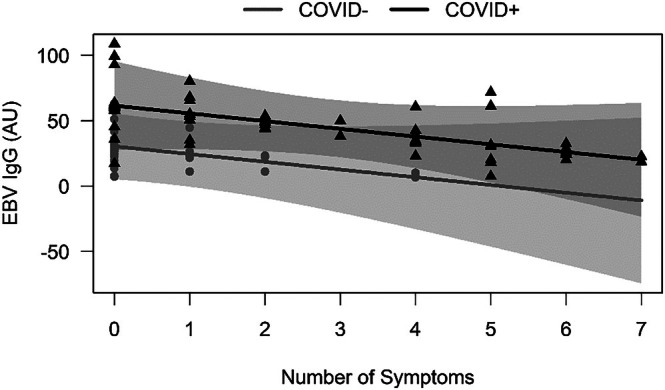
Predicted association between COVID‐19 Symptoms and EBV Scatterplot of predicted association between, COVID‐19‐associated symptoms, EBV IgG relative concentration (AU), and COVID‐19 status. COVID+ represented in gray, COVID− in black. Shaded regions represent standard error.

Based on these results, we conducted a post hoc analysis to examine independent associations when removing PSS scores and the COVID‐19 × PSS‐10 interaction term (Table 3, Models 1 and 2). These models demonstrate an overall negative trend in which greater COVID‐19 symptoms are associated with lower EBV IgG. However, there was no evidence of an interaction between symptoms and COVID‐19 status, and model fits were poorer compared to prior models including PSS scores (Table 3, Figure 4).

**TABLE 3:** Post hoc mixed effects linear model fitted coefficients.

	Model 4	Post hoc Model 1	Post hoc Model 2
COVID‐19 (+/−)	20.17 (−48.99, 89.33)	31.11 (−6.51, 68.74)	39.12 (−2.80, 81.03)
Days postpartum	0.06 (−0.04, 0.17)	0.09 (−0.03, 0.21)	0.09 (−0.03, 0.21)
Days since enrollment	0.02 (−0.18, 0.23)	0.03 (−0.18, 0.24)	0.02 (−0.18, 0.24)
Number of symptoms	−6.97 (−14.42, 0.49)	−5.91 (−14.79, 2.97)	3.58 (−20.64, 27.80)
COVID‐19 × symptoms	—	—	−10.81 (−36.50, 14.89)
Stress (avg. PSS score)	4.03 (−0.76, 8.83)	—	—
Stress × Covid‐19	1.88 (−3.75, 7.50)	—	—
AIC	1008.66	1025.806	1020.118

*Note:* 95% confidence intervals included in parentheses. All results are nonsignificant. Coefficients and confidence intervals are rounded to two decimal places to make it clear when confidence intervals for small coefficients cross zero. Rounding does not necessarily indicate the extent of precision.

As an additional post hoc analysis, we ran generalized additive mixed models (GAMM) to assess whether EBV IgG varied nonlinearly over time by COVID‐19 status. Smooth non‐linear terms for time were nonsignificant for both COVID+ and COVID− groups (*p* = 0.44 and *p* = 0.55, respectively, Table S5), and the fitted smooth term for COVID+ participants was approximately linear over time (Figure S3), suggesting that the linearity assumption of our primary analysis was reasonable for this group. Mean PSS‐10 score remained a significant predictor of EBV IgG (*β* = 4.93, *p* = 0.0005), consistent with our primary analysis.

## 4 Discussion

In this sample of postpartum women, EBV IgG relative concentration was higher in relation to perceived stress, but did not appear to be significantly elevated in relation to COVID‐19 infection. However, SARS‐CoV‐2 infections in this group were fairly mild (Martin et al. 2022). The variation in EBV IgG levels that we observed within our participants likely reflects their normal level of latent EBV expression, though on average these levels varied in relation to stress levels. Yet there were not consistent elevations that could indicate immunosuppression related to COVID‐19 status.

The association between stress and EBV IgG may have been more pronounced in COVID‐19+ mothers (Table 2, Model 3, Figure 3), as the inclusion of this interaction term improved overall model fit. However, the interaction coefficient was not statistically significant. In the full subsample of participants with PSS surveys (*n* = 64), COVID+ participants had somewhat higher PSS scores than COVID‐ participants shortly after enrollment (Figure 1), when they would have recently learned they were COVID+. However, their stress levels decreased over time, on average, becoming more similar to the COVID‐ group by 60 days after enrollment. The lack of statistical significance for the interaction term may reflect the smaller sample size of participants with viable HS samples, or variation in stress over time that is not captured in our model. The same could be said for the interaction of COVID‐19 status and symptoms, which was investigated post hoc. In Figures 3 and 4, the average predictive effects of stress and symptoms on EBV IgG, respectively, are parallel, but average EBV IgG relative concentration is higher for the COVID+ group in both plots. This suggests that COVID‐19 status might be a useful precision variable in analysis of the relations among stress, symptoms, and EBV IgG, or the immunomodulation that underlies EBV IgG relative concentration.

Perhaps COVID‐19 status *was* associated with EBV IgG and/or stress in some complex way not thoroughly measured in this exploratory analysis. Participant PSS scores were primarily low or moderate, despite potentially stressful changes in infant feeding, care, and household socialization due to pandemic restrictions and/or maternal infection. It is possible that the relatively privileged socioeconomic status of most participants buffered them somewhat from exceptional pandemic‐related stress, so we may have primarily observed normal fluctuations in EBV IgG expression related to typical amounts of stress. It is also possible that the PSS‐10 questionnaire may not have captured the psychosocial stress experienced by participants specifically related to the COVID‐19 pandemic or related to postpartum recovery. When the participant with high outlier data was excluded from analyses, it appeared to reduce confounding among COVID‐19, stress, and EBV relative concentration, and strengthened the evidence that stress influenced EBV relative concentration (see Table S7 and associated discussion for more details). Yet, given the small sample size and limited range of variation among participants, we did not exclude the outlier participant from our main analysis. Nonetheless, these results support the need for further research with larger sample sizes including more variance in stress and COVID‐19 case severity. Other methods for measuring stress should also be considered.

Pandemic‐related stressors were uniquely impactful and may have overshadowed other typical stressors and even COVID‐19 status itself. Previous studies found that pandemic‐related social restrictions were challenging and stressful for families with young children (Fong and Iarocci 2020; Spinelli et al. 2020). Still, isolation in the home came with unique trade‐offs. For example, a study of US families found that while physical activity decreased, and screen time and snack food consumption increased, families also ate more home‐cooked meals together (Carroll et al. 2020). While the COVID‐19 pandemic was a cause for unique fears and challenges which negatively impacted the mental health of parents, it also inspired lifestyle changes which may have had mixed impacts on mental health.

Only a minority of participants were leaving home for work or social activities at enrollment. A greater proportion were leaving home for work and social activities at 2 months post‐enrollment, and the change over time was more pronounced in the COVID+ group. This is consistent with previous research showing that most families stayed home and altered aspects of their routine during the pandemic (Fong and Iarocci 2020; Spinelli et al. 2020). The limited social activity outside the home had unknown effects on participants' mental health. COVID+ mothers also made temporary adjustments to their infant feeding practices, which may have increased feeding burdens, but these changes did not appear to substantially reduce breastfeeding frequency over time or in comparison with COVID− mothers.

One previous study observed that severe cases of COVID‐19 are associated with higher EBV IgG expression, while this association was not observed in milder cases of COVID‐19 (Chen et al. 2021). This motivated our investigation of symptoms as a proxy for severity. The negative association between COVID‐19 symptoms and EBV IgG relative concentration (AU) (Models 4) was unexpected, given the positive association we previously observed between maternal symptoms and anti‐SARS‐CoV‐2 antibodies (Martin et al. 2022). It is possible that SARS‐CoV‐2 infection compromised antibody production enough that anti‐EBV VCA IgG production did not rise in response to EBV viral expression, but COVID‐19 cases among participants were mild. Differences in relative infection severity and immune activity may not have been great enough to extend to variability in EBV levels. The overall nonsignificant negative association of COVID‐19 symptoms with EBV levels also did not appear to vary among infected and non‐infected mothers, which may reflect shared variance among asymptomatic COVID+ and symptomatic COVID− participants. For example, the participant showing the highest EBV IgG levels throughout follow‐up (+3 SD above sample mean) was COVID+ but reported no symptoms.

We did not observe a significant linear effect of time since enrollment or time postpartum on EBV IgG. It may be that there is a nonlinear association among these variables that is not captured by our analysis. While the minimal amount of EBV research in postpartum women has produced mixed results, one study demonstrated a slow return to latent baseline levels following EBV reactivation during pregnancy (Haeri et al. 2010). Other research has shown an average decrease during pregnancy (Amino et al. 1978; Malek et al. 1996). Another study found EBV IgG decreased from the first to third trimesters, and subsequently increased during the first 5 weeks postpartum, though it is not entirely clear whether this decrease was due to pregnancy‐related suppression of antibody production or viral expression (Christian et al. 2012). In this study, we observed the highest mean EBV IgG relative concentrations (AU) among participants who were more than a year postpartum, compared to those who were less than 6 months postpartum or 6 months–1 year (Table S4). However, the difference of these means should be interpreted cautiously due to large standard error and small and unequal sample sizes across postpartum groups.

Our study was limited by several methodological constraints. EBV IgG relative concentrations (AU) were estimated against a standard curve made from serial dilutions of neat Diamedix low positive IgG control because we lacked comparable seropositive blood samples of known concentration. Our results cannot be compared directly to EBV IgG concentrations from other studies. Due to the optical density differences in samples prepared from serum vs. HS, we could not assign positive/negative EBV status to samples from assay kit calibrator ranges and had no comparable blood samples with known EBV seropositivity from which we could prepare HS‐specific calibrators. While the resulting relative concentrations were internally consistent, we were not able to directly assess whether mothers were experiencing EBV reactivation.

Additionally, our sample size was small given that only a subset (*n* = 45 participants; 104 samples) of the original samples had sufficient blood volume remaining for EBV analysis, so subtle differences between groups may not have been detected (Martin et al. 2022; Pace et al. 2021). Most participants self‐identified as White (Non‐Hispanic), so a lack of racial or ethnic diversity limits the generalizability of our findings. Our conclusions are further limited since we used a count of COVID‐19 symptoms at recruitment as a proxy for case severity in our analysis, and cases were relatively mild.

Finally, aspects of our analytical protocol limit the interpretability of our results. Average reported stress was low among our participants, and this is one of the reasons we used numeric PSS‐10 scores rather than low/medium/high scores. This may limit the ability to compare our findings to other research, and future research in participants with higher stress and more severe cases of COVID‐19 may be needed to validate our findings. Finally, due to differences in survey collection between recruitment groups, we assessed associations between stress and EBV using overall mean PSS scores per participant rather than from each interview. This may have limited our ability to observe the effect of acute stress fluctuations on EBV IgG. Overall, our analyses rely on LME modeling, which assumes average linear associations among variables of interest and normal distribution of random effects and residual error. Since EBV IgG levels may be skewed and fluctuations over time are likely to be non‐linear, especially in COVID+ participants, LME modeling may minimize the appearance of true differences in EBV IgG between COVID+/− groups. However, our results were consistent with a post hoc nonlinear analysis (Supporting Information Section 5, Table S8). Further research should continue to investigate the complex potential relationships among latent EBV, stress, and SARS‐CoV‐2 infection, particularly in postpartum and pregnant women. Previous studies have investigated immune system modulation, stress, and EBV reactivation through methods which include salivary cortisol analysis, simulating stressful experiences and reported stressors, such as racial discrimination (Brook et al. 2017; Christian et al. 2012; Sausen et al. 2021; Zhu et al. 2013). Many of these methods could be used to further study factors which influence immune system modulation beyond what is covered in this exploratory analysis, and these methods could be helpful in expanding research in postpartum women. Though we did not find significant interactions between COVID‐19 and EBV IgG as a proxy for immunomodulation, the impacts of environmental stress and infectious disease could certainly impact pregnant and postpartum women differently and perhaps more strongly than other populations, beyond what is investigated here.

## 5 Conclusion

This study presents a novel analysis of associations among maternal SARS‐CoV‐2 infection, maternal stress, and EBV IgG expression during an unprecedented time in recent US history. Overall, our sample represents a population of postpartum women who were socially isolated to varying degrees over the course of follow‐up. Participants stayed home more than usual, leaving home primarily for work, and adjusted infant feeding behavior. Interactions among COVID‐19, stress, and symptoms did not have a statistically significant effect on EBV relative concentration. Yet the inclusion of interaction terms for these variables improved model fit. It may still be worth considering COVID‐19 status and symptoms in analyses related to EBV and stress. The effects of these variables on EBV IgG are likely somewhat interrelated, which may require a larger sample size with more extreme variation in infection and stress severity to disentangle. COVID‐19 status undoubtedly influenced symptoms in our participants, and it is plausible that COVID‐19 status also influenced stress levels. In this exploratory analysis, stress (measured by PSS‐10) was the strongest predictor of EBV IgG relative concentration, a proxy for immunomodulation. While COVID‐19 status and symptomology were not independently associated with EBV IgG relative concentration, COVID‐19 may be a useful precision variable to consider in addition to stress.

## Author Contributions

**Emma Anastasi:** conceptualization, data curation, formal analysis, methodology, visualization, writing – original draft preparation, writing – review and editing. **Delaney J. Glass:** investigation, writing – review and editing. **Tiffany D. Pan:** conceptualization, investigation, methodology, resources, writing – review and editing. **Eleanor Brindle:** conceptualization, funding acquisition, investigation, resources, writing – review and editing. **Beatrice Caffe:** conceptualization, data curation, investigation, writing – review and editing. **Caroline B. Smith:** data curation, writing – review and editing. **Amanda E. Kunkle:** data curation, writing – review and editing. **Maia A. Kent:** investigation, writing – review and editing. **Alexandra D. Navarrete:** investigation, writing – review and editing. **Christina D. W. Pace:** investigation, writing – review and editing. **Dan T. A. Eisenberg:** funding acquisition, methodology, writing – review and editing. **Ryan M. Pace:** conceptualization, data curation, funding acquisition, methodology, project administration, supervision, writing – review and editing. **Janet E. Williams:** conceptualization, funding acquisition, methodology, project administration, supervision, writing – review and editing. **Sylvia H. Ley:** conceptualization, funding acquisition, methodology, project administration, resources, supervision, writing – review and editing. **Celestina Barbosa‐Leiker:** conceptualization, funding acquisition, methodology, project administration, resources, supervision, writing – review and editing. **Mark A. McGuire:** conceptualization, funding acquisition, resources, writing – review and editing. **Michelle K. McGuire:** conceptualization, funding acquisition, methodology, project administration, resources, supervision, writing – review and editing. **Courtney L. Meehan:** conceptualization, funding acquisition, methodology, project administration, resources, supervision, writing – review and editing. **Melanie A. Martin:** conceptualization, data curation, formal analysis, funding acquisition, investigation, methodology, project administration, resources, supervision, writing – original draft preparation, writing – review and editing.

## Funding

This work was supported by the National Science Foundation (IOS‐BIO 2031753, IOS‐BIO 2031715, IOS‐BIO 2031888, IOS‐BIO 2031761), Bill and Melinda Gates Foundation (INV‐016943), Eunice Kennedy Shriver National Institute of Child Health and Human Development (P2C HD042828), Center for Studies in Demography and Ecology, University of Washington, Idaho Agricultural Experiment Station – Hatch project (IDA01643), National Institute of General Medical Sciences (P20GM152304), Department of Anthropology, University of Washington, and Health Equity Research Center, Washington State University.

## Ethics Statement

This study conforms to the Declaration of Helsinki and the US Federal Policy for the Protection of Human Subjects. The studies involving human participants were reviewed and approved by the institutional review boards of the University of Idaho (20‐056, 20‐060), the University of Washington (STUDY00010215), and Tulane University (2020‐602). Adults provided written and verbal consent for themselves and their minor children to participate in this study. The participants provided their written informed consent to participate in this study.

## Conflicts of Interest

The authors declare no conflicts of interest.

## Supporting information


**Data S1:** ajhb70289‐sup‐0001‐supinfo.docx.
**Figure S1:** PSS‐10 responses plotted by question and by days since enrollment.
**Figure S2:** Density plots of EBV IgG relative concentration (AU).
**Figure S3:** GAMM plots of nonlinear EBV IgG over time, with random slope and intercept for participants and COVID+/− groups.
**Table S1:** PSS scores by day of follow‐up for excluded participants versus overall sample.
**Table S2:** Summary statistics for PSS‐10 scores.
**Table S3:** Summary statistics for PSS‐10 responses by screening question.
**Table S4:** Descriptive statistics for EBV IgG concentration (AU).
**Table S5:** Fitted mixed effects linear models, including sensitivity analysis.
**Table S6:** Fitted mixed effects linear models with log transformation of EBV IgG.
**Table S7:** Fitted mixed effects linear models from additional sensitivity analysis focused on outlier participant.
**Table S8:** Fitted coefficients for smooth nonlinear EBV IgG regression over time by COVID‐19 status.

## Data Availability

The data that support the findings of this study are available on request from the corresponding author. The data are not publicly available due to privacy or ethical restrictions.
