# Supplementary material for: Relationships Among Maternal Epstein–Barr Virus Antibodies, COVID‐19, and Stress in Mothers up to 1‐Year Postpartum
Source: Am J Hum Biol. 2026 Jun 12;38(6):e70289. doi: 10.1002/ajhb.70289 (PMC13263636; doi:10.1002/ajhb.70289)

# Supporting Information

## Section 1: Materials and Methods

### Preparation of Controls & Calibrator on Hemaspot HF Devices

Gold Standard Diagnostics – Diamedix EBV VCA IgG Kit (Cat. # 720-600, Lot # 30502)

03/23/2023 Spot Kit Controls onto Hemaspot HF Devices

*Prepare washed red blood cells*

- Draw venous whole blood into EDTA tube (gently mix by inverting 6-10 times)
- Centrifuge for 15 minutes at ~1500 xg to separate plasma and buffy coat from red blood cells
- Discard plasma and buffy coat or aliquot and save for other purposes
- Transfer red blood cells to a new 15 mL centrifuge tube
- Wash
  - For a given volume of red blood cells, add an equal volume of saline (0.86 g NaCl / 100 ml ddH2O)
  - Rock on rotator for 5 minutes to mix
  - Centrifuge for 10-15 minutes at ~1300 xg
  - Remove and discard supernatant (saline)
- Repeat wash step two more times for a total of three washes

*Mix kit-provided controls and cut-off calibrator with washed red blood cells*

- Negative Control (Cat. # 720-603, Lot # 30522)
- Cut-Off Calibrator (Cat. # 720-604, Lot # 30532)
- Low Positive Control (Cat. # 720-605, Lot # 30542)
- Mix equal volumes (v/v) of controls and calibrator with red blood cells
  - Pipette 75 uL of washed red blood cells into each of three 1.5 mL tubes
  - Pipette 75 uL of each control or calibrator into the appropriately labeled tube
  - Rock on rotator for 15 minutes
- Drop controls and cut-off calibrator with RBC mixtures onto Hemaspot HF devices
  - Use wide-bore pipette tips with pipettor set to 50 uL
  - Dispense two “drops” on each Hemaspot HF device for a total of 100 uL on each device
  - Allow mixture to disburse and saturate the filter paper for 1 minute
- Close device clamshell and allow to dry at room temperature overnight
- Store frozen at -20 C

*Further Details:*

EBV IgG concentration was analyzed using a qualitative commercial enzyme immunoassay kit following a protocol previously validated for quantitative EBV IgG measurement from protein card DBS samples (Eick et al., 2016). The protocol was further adapted as follows: We made an 8-point standard curve for each plate using a serial dilution of neat Diamedix low positive IgG control (dilution factors for standards 1-8 as follows: neat, 1:4, 1:16, 1:32, 1:64, 1:128, 1:256, 1:1024). Eick and colleagues (2016) use a calibrator sourced from the Mikrogen recomWell EBV-VCA IgG kit (Biosell Solutions Inc., Las Vegas, NV), which is separate from the Diamedix kit used for the rest of their assay. No precise concentration is given for the Diamedix low positive control, so our quantitative results based on the known dilution factors in our standard curve are internally consistent but are expressed in arbitrary units. The serum-based kit-supplied cutoff calibrator served as our low positive control, eluted in buffer and washed red blood cells to mimic an HS sample. An in-house high positive control and the kit-supplied negative control were prepared in the same manner. All samples, standards, and controls were tested in duplicate. Similarly, Eick and colleagues (2016) use arbitrary units (U/mL) and set a seropositivity cutoff value of 21 U/mL based on the absorbance of their positive and negative controls.

All samples, standards, and controls were run in duplicate. The inter-assay CV, based on the low positive control, was 9.14, and based on the cutoff calibrator was 17.68. The intra-assay CV for each of three plates were 6.18%, 8.68% and 7.86%. We calculated upper and lower limits of quantification adding 2 standard deviations to the optical density of the most dilute standard in our curve on each plate and subtracting 2 standard deviations from the highest standard. All samples were within the limits of quantification (Plate 1: 0.071-1.238, Plate 2: 0.013-1.234, Plate 3: 0.017-1.429 (AU)). Due to sample and resource limitations, it was not possible to rerun samples with CVs > 15% between duplicates (n =15, CV range 15.13 - 36.87) per standard lab protocols. When compared to a cutoff calculated from the optical density of the cutoff calibrator, all positive controls tested positive, and negative controls and blanks tested negative.

Unlike Eick and colleagues (2016), we did not ultimately apply a cutoff value and classify samples as positive or negative, instead modelling relative concentration values among our participants with mixed-effects linear regression. While our goal was to analyze immunomodulation rather than positivity, we observed that most samples had higher relative concentration values than the cutoff calibrator which had been prepared to mimic a DBS sample as a low positive control.

#### Table S1: PSS scores by day of follow-up for excluded participants versus overall sample.

|  | **Overall (n=64)** | |
| --- | --- | --- |
|  | **Mean ± SD** | **Range** |
| Day 1 | 13 ± 7 | 0-29 |
| Day 7 | 11 ± 7 | 1-30 |
| Day 14 | 9 ± 8 | 0-32 |
| Day 21 | 9 ± 6 | 0-25 |
| Day 30 | 8 ± 7 | 0-24 |
| Day 60 | 10 ± 7 | 0-31 |

## Section 2: Ten-Question Perceived Stress Screening (PSS-10)

For all of the below questions, available answers were: Never (0), Almost Never (1), Sometimes (2), Fairly Often (3), Very Often (4). Because of the phrasing of the questions, before summing responses to find the overall score, you must reverse the scores for questions 4, 5, 7, and 8. On these 4 questions, change the scores as follows:

0 = 4, 1 = 3, 2 = 2, 3 = 1, 4 = 0.

Then sum the scores for each question. Scores can range from 0 to 40 with higher scores indicating higher perceived stress. Scores from 0-13 would be considered low stress. Scores from 14-26 indicate moderate stress. Scores from 27-40 indicate high perceived stress.

Full text of screening:

1. In the last week, how often have you been upset because of something that happened unexpectedly?
2. In the last week, how often have you felt that you were unable to control the important things in your life?
3. In the last week, how often have you felt nervous and "stressed"?
4. In the last week, how often have you felt content about your ability to handle your personal problems?
5. In the last week, how often have you felt that things were going your way?
6. In the last week, how often have you found that you could not cope with all the things that you had to do?
7. In the last week, how often have you been able to control irritations in your life?
8. In the last week, how often have you felt that you were on top of things?
9. In the last week, how often have you been angered because of things that were outside of your control?
10. In the last week, how often have you felt difficulties were piling up so high that you could not overcome them?

#### Table S2: Summary Statistics for PSS-10 Scores

|  | **Overall (n=64)** | | **COVID+ (n=37)** | | **COVID- (n=27)** | |
| --- | --- | --- | --- | --- | --- | --- |
|  | **Mean ± SD** | **Range** | **Mean ± SD** | **Range** | **Mean ± SD** | **Range** |
| Day 1 | 13 ± 7 | 0-29 | 15 ± 7 | 1-29 | 10 ± 6 | 0-21 |
| Day 7 | 11 ± 7 | 1-30 | 11 ± 8 | 1-30 | 9 ± 4 | 1-16 |
| Day 14 | 9 ± 8 | 0-32 | 9 ± 8 | 0-32 | - | - |
| Day 21 | 9 ± 6 | 0-25 | 9 ± 7 | 0-25 | 10 ± 6 | 2-23 |
| Day 30 | 8 ± 7 | 0-24 | 8 ± 7 | 0-24 | - | - |
| Day 60 | 10 ± 7 | 0-31 | 10 ± 9 | 0-31 | 5 ± 1 | 1-21 |

#### Table 3: Summary Statistics for PSS-10 Responses by Screening Question

Coding has already been reversed for questions 4,5,7, and 8.

| **Question** | **Mean** | **Std Dev** | **Range** |
| --- | --- | --- | --- |
| 1 | 1.19 | 1.11 | 0-4 |
| 2 | 0.99 | 1.14 | 0-4 |
| 3 | 1.8 | 1.11 | 0-4 |
| 4 | 0.59 | 0.77 | 0-4 |
| 5 | 1.07 | 0.97 | 0-4 |
| 6 | 0.94 | 0.96 | 0-4 |
| 7 | 1.03 | 0.88 | 0-4 |
| 8 | 1.16 | 0.86 | 0-4 |
| 9 | 1.22 | 1.1 | 0-4 |
| 10 | 0.7 | 0.98 | 0-4 |

#### Figure S1: PSS-10 Responses Plotted by Question and by Days Since Enrollment

Plots of PSS scores by question, one plot per number of days since enrollment. Coding has already been reversed for questions 4, 5, 7, and 8. Responses were whole numbers between 0 and 4, equating to “Never,” “Almost Never,” “Sometimes,” “Fairly Often,” and “Often.” Horizontal and vertical jitter has been added to plotted points for visibility.

 
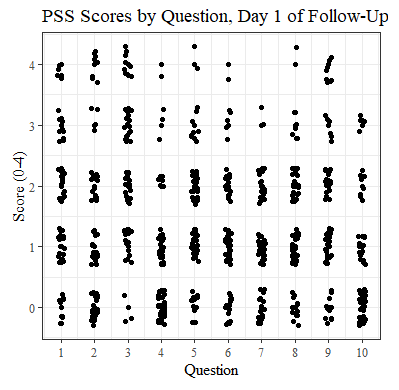

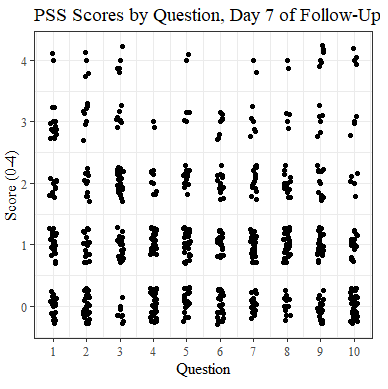


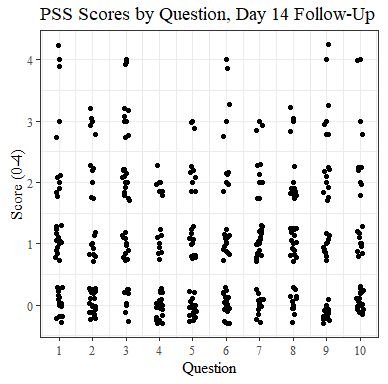

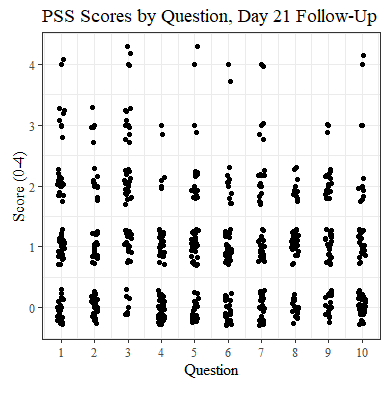


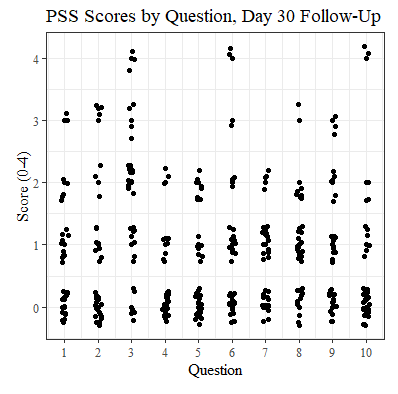

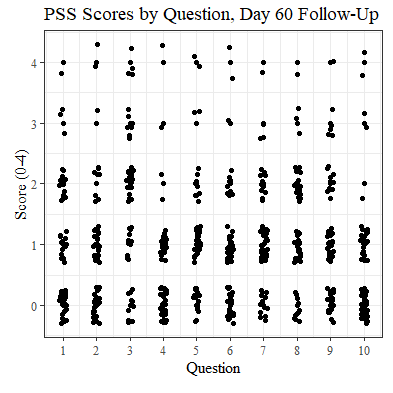


## Section 3: Summary of EBV IgG Assay Results

#### Figure S2: Density plots of EBV IgG Relative Concentration (AU)

Overall distribution (left) and distributions for COVID+/- groups separated (right)

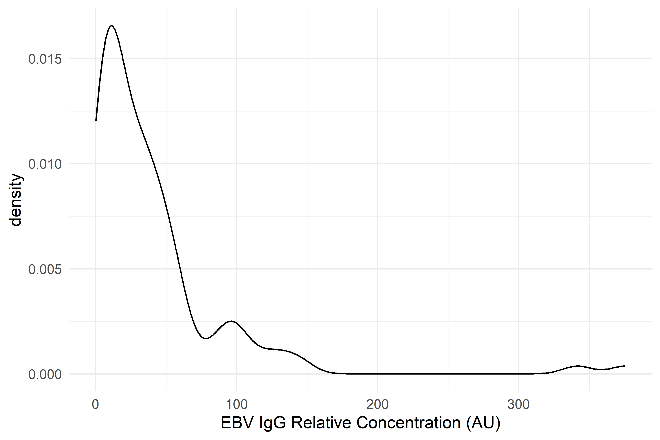

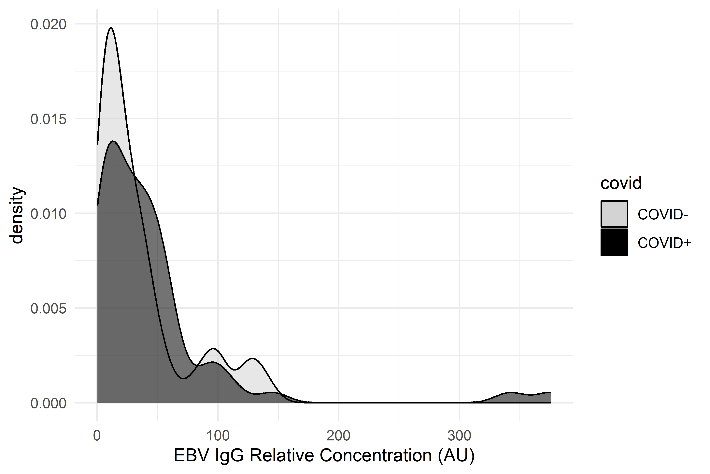


#### Table S4: Descriptive Statistics for EBV IgG Concentration (AU)

| **Subsample** | **Mean ± SD** | **Range** | **N Participants (N Samples)** |
| --- | --- | --- | --- |
| Full Sample | 40.58 ± 55.71 | 0.25 - 375.46 | 45 (104) |
| **COVID-19 status** | | | |
| COVID + | 45.19± 65.68 | 0.25 - 375.46 | 25 (61) |
| COVID – | 34.03 ± 37.04 | 1.61 - 139.33 | 20 (43) |
| **Study Day** | | | |
| Day 14 | 30.21 ± 30.93 | 0.25 - 145.72 | 35 (35) |
| Day 21 | 53.08 ± 40.96 | 2.67 - 112.00 | 8 (8) |
| Day 30 | 47.31 ± 70.42 | 1.244 - 375.46 | 31 (31) |
| Day 60 | 42.38 ± 64.35 | 2.95 - 341.76 | 30 (30) |
| **Postpartum period (days)** | | | |
| < 6 months | 23.98 ± 17.71 | 0.25 - 83.84 | 17 (38) |
| 6 months - 1 year | 44.25 ± 38.61 | 3.28 - 145.72 | 20 (46) |
| > 1 year | 63.68 ± 107.40 | 2.67 - 375.46 | 8 (20) |
| **COVID-19 symptoms**  (COVID+ only; n participants = 25; n samples = 61) | | | |
| 0 | 83.55 ± 115.17 | 2.67- 375.46 | 6 (16) |
| 1 | 44.83 ± 23.39 | 5.79 – 83.84 | 4 (8) |
| 2 | 17.49 ± 12.28 | 3.98 - 33.01 | 3 (8) |
| 3 | 37.91 ± 8.87 | 31.64 - 44.18 | 1 (2) |
| 4 | 30.04 ± 16.44 | 9.90 - 55.71 | 3 (8) |
| 5 | 51.32 ± 36.71 | 0.25 - 112.00 | 4 (9) |
| 6 | 12.75 ± 9.93 | 4.51 - 33.33 | 3 (8) |
| 7 | 20.66 ± 2.51 | 18.89 - 22.44 | 1 (2) |

Symptoms recorded were cough, fever, fatigue, difficulty breathing, diarrhea, sore throat, stuffy/runny nose, headache, itchy/watery eyes, sneezing, stomach pain, loss of smell/taste, heart-related issues, dizziness/lightheadedness, body aches, muscle aches, joint pain, back pain, tingling in arms/legs, thrush, Hashimoto’s thyroiditis, gurgly stomach, chest tightness, postnasal drip, rash, nausea, vomiting, eye pressure, scratchy throat, sweating, chills, foggy brain, burning sensation in nose, malaise, loss of appetite, congestion, vertigo, pink eye, hair loss, rash in mouth/throat, whiteness on tongue, shortness of breath, rapid heartbeat, pneumonia, nosebleed, insomnia, asthma, sinus burning, stinging eyes, heart palpitations, dyspnea on exertion, weird taste in mouth, ears popping with swallowing, ears plugged up, and night waking.

## Section 4: Mixed Effects Linear Modeling

#### Table S5: Fitted Mixed Effects Linear Models, Including Sensitivity Analysis

Models indicated by rows, variables indicated by columns, fitted coefficients in table cells and 95% confidence intervals shown in parentheses. Bolding indicates coefficients and confidence intervals notably different with the inclusion of high-CV samples (only occurs in Model 2).

| **Mixed Effects Linear Model Fitted Coefficients**  Confidence Intervals included in parentheses | | | | | |
| --- | --- | --- | --- | --- | --- |
|  | **Model 1** | **Model 1 (exclude high-CV samples)** | **Model 2** | **Model 2 (exclude high-CV samples)** |  |
| **COVID-19 (+/-)** | 17.72 (-14.69, 50.15) | 19.52 (-10.63, 49.72) | **24.24 (-3.68, 52.16)** | **26.78 (1.33, 52.25)** |  |
| **Days Postpartum** | 0.11 (-0.01, 0.23) | 0.09 (-0.02, 0.21) | 0.10 (-0.01, 0.20) | 0.08 (-0.01, 0.17) |  |
| **Days Since Enrollment** | 0.03 (-0.18, 0.24) | -0.02 (-0.25, 0.22) | 0.02 (-0.18, 0.23) | -0.03 (-0.25, 0.21) |  |
| **Stress**  **(Avg. PSS Score)** | - |  | 5.22 (2.64, 7.80) | 5.06 (2.71, 7.41) |  |
| **Stress x Covid-19** | - |  | - | - |  |
| **Symptoms** | - |  | - | - |  |
| **AIC** | 1030.33 | 883.30 | 1016.88 | 868.53 |  |

| **Mixed Effects Linear Model Fitted Coefficients Continued**  Confidence Intervals included in parentheses | | | | |
| --- | --- | --- | --- | --- |
|  | **Model 3** | **Model 3 (exclude high-CV samples)** | **Model 4** | **Model 4 (exclude high-CV samples)** |
| **COVID-19 (+/-)** | 0.14 (-68.05, 68.33) | 0.13 (-61.30, 61.60) | 20.17 (-48.99, 89.33) | 17.36 (-45.09, 79.84) |
| **Days Postpartum** | 0.09 (-0.01, 0.20) | 0.07 (-0.02, 0.17) | 0.06 (-0.04, 0.17) | 0.05 (-0.04, 0.14) |
| **Days Since Enrollment** | 0.02 (-0.18, 0.23) | -0.02 (-0.25, 0.21) | 0.02 (-0.18, 0.23) | -0.02 (-0.24, 0.22) |
| **Stress**  **(Avg. PSS Score)** | 3.58 (-1.37, 8.53) | 3.25 (-1.21, 7.72) | 4.03 (-0.76, 8.83) | 3.62 (-0.72, 7.95) |
| **Stress x Covid-19** | 2.25 (-3.57, 8.07) | 2.50 (-2.76, 7.76) | 1.88 (-3.75, 7.50) | 2.15 (-2.96, 7.25) |
| **Symptoms** | - | - | -6.97 (-14.42, 0.49) | -6.06 (-12.86, 0.77) |
| **AIC** | 1014.26 | 865.84 | 1008.66 | 860.72 |

#### Table S6: Fitted Mixed Effects Linear Models with Log Transformation of EBV IgG

Models indicated by rows, variables indicated by columns, fitted coefficients in table cells and 95% confidence intervals shown in parentheses.

| **Mixed Effects Linear Model Fitted Coefficients**  Confidence Intervals included in parentheses | | | | |
| --- | --- | --- | --- | --- |
|  | **Model 1** | **Model 1 (log(EBV))** | **Model 2** | **Model 2 (log(EBV))** |
| **COVID-19 (+/-)** | 17.72 (-14.69, 50.15) | 0.19 (-0.53, 0.91) | 24.24 (-3.68, 2.16) | 0.29 (-0.40, 0.97) |
| **Days Postpartum** | 0.11 (-0.01, 0.23) | 0.00 (0.00, 0.00) | 0.10 (-0.01, 0.20) | 0.00 (0.00, 0.00) |
| **Days Since Enrollment** | 0.03 (-0.18, 0.24) | 0.00 (0.00, 0.01) | 0.02 (-0.18, 0.23) | 0.00 (0.00, 0.01) |
| **Stress**  **(Avg. PSS Score)** | - | - | **5.22 (2.64, 7.80)**  **p = 0.0004** | **0.07 (0.01, 0.14)**  **P = 0.03** |
| **Stress x Covid-19** | - | - | - | - |
| **Symptoms** | - | - | - | - |
| **AIC** | 1030.33 | 265.24 | 1016.88 | 267.44 |

| **Mixed Effects Linear Model Fitted Coefficients**  Confidence Intervals included in parentheses | | | | |
| --- | --- | --- | --- | --- |
|  | **Model 3** | **Model 3 (log(EBV))** | **Model 4** | **Model 4 (log(EBV))** |
| **COVID-19 (+/-)** | 0.14 (-68.05, 68.33) | 0.70 (-0.98, 2.37) | 20.17 (-48.99, 89.33) | 1.06 (-0.66, 2.79) |
| **Days Postpartum** | 0.09 (-0.01, 0.20) | 0.00 (0.00, 0.00) | 0.06 (-0.04, 0.17) | 0.00 (0.00, 0.00) |
| **Days Since Enrollment** | 0.02 (-0.18, 0.23) | 0.00 (0.00, 0.01) | 0.02 (-0.18, 0.23) | 0.00 (0.00, 0.01) |
| **Stress**  **(Avg. PSS Score)** | 3.58 (-1.37, 8.53) | 0.10 (-0.02, 0.22) | 4.03 (-0.76, 8.83) | 0.11 (-0.01, 0.23) |
| **Stress x Covid-19** | 2.25 (-3.57, 8.07) | -0.04 (-0.18, 0.10) | 1.88 (-3.75, 7.50) | -0.05 (-0.31, 0.06) |
| **Symptoms** | - | - | -6.97 (-14.42, 0.49) | -0.13 (-0.19, 0.09) |
| **AIC** | 1014.26 | 272.51 | 1008.66 | 275.66 |

#### Table S7: Fitted Mixed Effects Linear Models from Additional Sensitivity Analysis Focused on Outlier Participant

|  | **Model 1** | **Model 1**  **(Excluding Participant 355)** | **Model 2** | **Model 2**  **(Excluding**  **Participant 355)** |
| --- | --- | --- | --- | --- |
| **COVID-19 (+/-)** | 17.72 (-14.69, 50.15) | 1.99 (-16.34, 0.34) | 24.24 (-3.68, 52.16) | 6.09 (-11.57, 23.75) |
| **Days Postpartum** | 0.11 (-0.01, 0.23) | 0.00 (-0.07, 0.07) | 0.10 (-0.01, 0.20) | 0.01 (-0.06, 0.07) |
| **Days Since Enrollment** | 0.03 (-0.18, 0.24) | 0.03 (-0.18, 0.23) | 0.02 (-0.18, 0.23) | 0.02 (-0.18, 0.22) |
| **Stress**  **(Avg. PSS Score)** | - | - | 5.22 (2.64, 7.80)  **p = 0.0004** | 2.00 (0.25, 3.76)  **(p = 0.03)** |
| **Stress x Covid-19** | - | - | - | - |
| **Number of Symptoms** | - | - | - | - |
| **AIC** | 1030.33 | 960.39 | 1016.88 | 956.16 |

|  | **Model 3** | **Model 3**  **(Excluding**  **Participant 355)** | **Model 4** | **Model 4**  **(Excluding**  **Participant 355)** |
| --- | --- | --- | --- | --- |
| **COVID-19 (+/-)** | 0.14 (-68.05, 68.33) | 26.98 (-15.37, 69.33) | 20.17 (-48.99, 89.33) | 34.82 (-8.58, 78.26) |
| **Days Postpartum** | 0.09 (-0.01, 0.20) | 0.01 (-0.06, 0.07) | 0.06 (-0.04, 0.17) | 0.00 (-0.07, 0.07) |
| **Days Since Enrollment** | 0.02 (-0.18, 0.23) | 0.02 (-0.19, 0.22) | 0.02 (-0.18, 0.23) | 0.02 (-0.19, 0.22) |
| **Stress**  **(Avg. PSS-10 Score)** | 3.58 (-1.37, 8.53) | 3.36 (0.32, 6.40)  **(p = 0.04)** | 4.03 (-0.76, 8.83) | 3.56 (0.56, 6.57)  **(p = 0.03)** |
| **Stress x Covid-19** | 2.25 (-3.57, 8.07) | -2.00 (-5.69, 1.69) | 1.88 (-3.75, 7.50) | -2.98 (-7.71, 1.75) |
| **Number of Symptoms** | - | - | -6.97 (-14.42, 0.49) | -2.04 (-5.67, 1.58) |
| **AIC** | 1014.26 | 953.93 | 1008.66 | 950.86 |

### Discussion:

One participant had an average EBV relative concentration of 358.61 AU across follow-up. This is twice as high as the next highest EBV result (145.72 AU) and 10 times as high as the average relative concentration for all other participants (34.34 AU). The outlier participant had an average total PSS-10 score of 25, indicating moderate stress, which was higher than the next highest PSS-10 score from all other participants (total score of 19, indicating moderate stress). The average PSS-10 score for all other participants was 10, indicating low stress. The outlier participant did not report any COVID-19 symptoms. Overall, this participant likely experienced high stress due to factors other than SARS-CoV-2 infection, and this stress likely contributed to high EBV IgG relative concentrations across follow-up.

The coefficient on COVID-19 status was not significant across models, regardless of whether the outlier participant was included or excluded. With the outlier participant included, coefficients on the interaction of COVID-19 and stress were positive, yet when this participant’s data were excluded the coefficient on this interaction was negative (though these coefficients are not statistically significant). This in addition to the strengthened association between stress and EBV IgG across models 2, 3, and 4 suggests reduced confounding when the outlier participant was excluded.

The outlier participant clearly had undue influence on our results, given the observed differences in model results, but this individual is a true outlier in the sense that we have no reason to suspect their scores are erroneous, particularly because they had consistently higher EBV relative concentration than other participants. This participant's results speak to the need for a larger sample size with more variance that would include more severe cases of COVID-19, people with higher stress, and that may include people with higher EBV antibody concentrations as well.

**Section 5: Generalized Additive Mixed Modeling (GAMM)**

As a post hoc analysis, we used generalized additive mixed modeling (GAMM) to model EBV IgG relative concentration as non-linear over time, while allowing associations over time to differ by COVID-19 status, with mean stress and days postpartum as linear covariates. We allowed for random variation in slope and intercept by participant to account for non-random correlation among observations from the same participant. Smooth non-linear terms for COVID+ and COVID- over time were not statistically significant (p = 0.44 and p = 055, respectively), and the fitted smooth term for COVID+ participants was approximately linear over time (Figure S3). In addition to smooth non-linear terms, this analysis produces fitted coefficients akin to the results of linear mixed effects modelling, shown below:

**Table S8: Fitted Coefficients for Smooth Non-Linear EBV IgG Regression Over Time by COVID-19 Status**

|  | **Estimate** | **P-Value** |
| --- | --- | --- |
| **Stress**  **(Avg. PSS-10 Score)** | **4.93** | **0.0005** |
| **Days Postpartum** | 0.08 | 0.14 |
| **Days Since Enrollment * COVID-** | 3.64 | 0.24 |
| **Days Since Enrollment * COVID+** | -1.64 | 0.55 |

Figure S3: GAMM plots of non-linear EBV IgG over time, with random slope and intercept for participants and COVID+/- groups


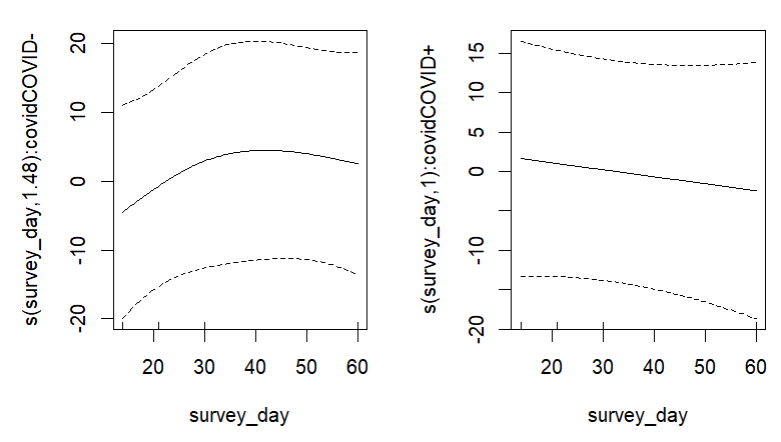

Supplement: Supplementary file 1 — Data S1: ajhb70289‐sup‐0001‐supinfo.docx. Figure S1: PSS‐10 responses plotted by question and by days since enrollment. Figure S2: Density plots of EBV IgG relative concentration (AU). Figure S3: GAMM plots of nonlinear EBV IgG over time, with random slope and intercept for participants and COVID+/− groups. Table S1: PSS scores by day of follow‐up for excluded participants versus overall sample. Table S2: Summary statistics for PSS‐10 scores. Table S3: Summary statistics for PSS‐10 responses by screening question. Table S4: Descriptive statistics for EBV IgG concentration (AU). Table S5: Fitted mixed effects linear models, including sensitivity analysis. Table S6: Fitted mixed effects linear models with log transformation of EBV IgG. Table S7: Fitted mixed effects linear models from additional sensitivity analysis focused on outlier participant. Table S8: Fitted coefficients for smooth nonlinear EBV IgG regression over time by COVID‐19 status. [file AJHB-38-e70289-s001.docx]
